# Unravelling interdisciplinary interactions and researcher migration across the evolution of perovskite solar cell research through comprehensive bibliometrics

**DOI:** 10.1098/rsos.241986

**Published:** 2025-06-04

**Authors:** Jun-Seok Yeo, A-Ram Kim

**Affiliations:** ^1^ Korea Institute of Science and Technology Evaluation and Planning, Maengdong-myeon, The Republic of Korea

**Keywords:** perovskite solar cell, organic solar cell, dye-sensitized solar cell, next-generation solar cell, bibliometric analysis

## Abstract

From 2009 to 2023, over 20 000 papers have been published on perovskite solar cells (PeSCs), highlighting the significant academic interest and research activity in this field. This exponential growth in research stems from the integration of various established research areas. Additionally, advancements in PeSCs have had a reciprocal influence on other technologies. Therefore, understanding the inflows and outflows of knowledge across the PeSC field is essential to fully grasp its intellectual landscape. However, no systematic bibliometric analyses have been conducted to address these dynamics. In this study, we performed a comprehensive bibliometric analysis to examine the development and thematic evolution of PeSC research. Our approach included analysing citation relationships, tracking publication trends in PeSC-related fields, identifying highly cited papers (HCPs) and mapping keywords and collaboration networks. A key aspect of our study was identifying researchers who have made significant contributions to the PeSC research community. We initially identified individuals who published 10 or more HCPs between 2009 and 2021, classifying them as PeSC highly cited researchers (PeSC-HCRs). We then employed social network analysis to explore their research activities from 2005 to 2022, using author keywords to track thematic trends. To ensure a structured analysis, we categorized all publications by publication year and InCites citation topics, allowing us to detect shifts in research priorities and the emergence of new subdisciplines over time. Furthermore, we investigated evolving patterns of collaboration among PeSC-HCRs, providing insights into how collaboration networks have influenced the development and diversification of the PeSC field. This analytical approach offers a novel perspective on the study of emerging technologies and is the first of its kind applied to PeSC research. The insights gained from this study can serve as a foundation for forecasting the developmental trajectories of other emerging technologies in the future.

## 1. Introduction

Hybrid organic–inorganic perovskite solar cells (PeSCs) have emerged as a disruptive technology in the field of next-generation photovoltaics (PVs), particularly in comparison with organic solar cells (OSCs) and dye-sensitized solar cells (DSSCs). Organometallic perovskites, with the chemical formula ABX_3_, possess exceptional photoactive properties, such as a high absorption coefficient, and a tunable bandgap through stoichiometric control [1]. Additionally, their low exciton binding energy leads to long charge-carrier lifetimes, extended diffusion lengths and high defect tolerance [1,2]. PeSCs also offer notable advantages, including lightweight design, mechanical flexibility and the potential for scalable and low-temperature manufacturing processes [3,4]. A pivotal study by Miyasaka *et al*. in 2009 triggered a surge of research on PeSCs [5], resulting in over 20 000 publications on the topic from 2009 to 2023 [6,7]. This extensive research effort has driven an impressive improvement in power conversion efficiency (PCE), increasing from 3.8 to 26.7% within just 14 years [8–10], nearly matching the crystalline silicon (c-Si) record of 26.8% [10]. This achievement solidifies the potential of PeSCs as a viable alternative for disrupting the c-Si-based PV market. Furthermore, this rapid progress has catalysed a shift in research priorities within next-generation PVs, with increasing focus on PeSCs at the expense of OSCs and DSSCs. For certified efficiency, the record-breaking cycle, that is, the frequency at which new efficiency records are being set or broken within a specific timeframe, for OSCs and DSSCs is increasing, particularly for DSSCs, which have not broken the record since achieving 13.0% efficiency in 2020 [10,11]. Additionally, bibliometric studies indicate that the number of annual publications on OSCs and DSSCs has gradually declined since their peaks in 2014–2015 [12,13]. Conversely, new high PeSC PCE records have been frequently reported regardless of the device architecture; notably, recent updates have been driven by the private sector, such as Oxford PV and LONGi [10]. The growth rate in the number of papers published per year has recently reached saturation; however, the number of annual publications still exceeds 3000 [14]. This evolution in research gives rise to the question of whether the advent of PeSCs has significantly influenced the transition in research focus within the field of next-generation PVs.

In addition to the macro-trends evident in the statistical data, the subtopics within the PeSC field have consistently evolved over time. In the early stages of research, improving device efficiency was the primary focus. However, other critical factors for the successful deployment of PeSCs, such as stability, lead-free materials, flexible devices and large-scale manufacturing, have gained increasing attention [2,4,15–18]. Our previous bibliometric study objectively verified that the proportion of publications on stability and public acceptability increased significantly, while those focused on efficiency decreased [19]. More importantly, the results of topic modelling revealed that themes related to scaling-up, stability and public acceptability became more prominent over time.

During this development, contributions from multiple disciplines were integrated into the development of PeSC technology. Particularly, in the early stages, the influx of research findings and researchers from other fields played crucial roles in the development of PeSCs. Additionally, PeSC research has influenced advancements in other fields utilizing perovskite materials. Hence, extensive interdisciplinary collaboration has enriched the technological progress of PeSCs and expanded the horizon of perovskite material applications ranging from light-emitting diodes (LEDs) to advanced sensing technologies [20,21]. This cross-disciplinary is largely attributed to the intrinsic connections between PeSCs and diverse scientific fields, such as inorganic chemistry, organic chemistry, physics, photonics, electronics and materials science. Therefore, analysing the inflows and outflows of research across the PeSC field is essential to fully understand its intellectual structures.

Recently, several bibliometric studies on PeSCs have been conducted. Shikoh *et al*. analysed PeSC papers published from 2009 to 2019 using various bibliometric methods to assess influential papers, institutions, countries and authors with a focus on citations [14]. Fonteyn *et al.* provided an overview of topics in the academic literature on OSCs and PeSCs from 2008 to 2017, evaluating how these topics evolved over time [12]. Kumar *et al.* examined 11 092 articles from the Scopus and Web of Science databases, exploring trends from the inception of PeSC research, key contributors (including countries, researchers and sources), detailed keyword analysis and thematic trends [22]. While these studies offer valuable insights into the evolution of PeSC technology and identify the most influential researchers and institutions, they are primarily limited to the analysis of the PeSC field itself. Although these bibliometric analyses contribute to mapping the development of PeSCs, they do not fully capture the dynamic inflows and outflows of research between PeSCs and other scientific domains.

As the field of technology evolves, it is shaped not only by internal advancements but also by adjacent research areas and the social structures that govern the spread of knowledge [23–25]. In this context, understanding these knowledge structures and networks that facilitate the dissemination of information is crucial for analysing the trajectory of an emerging field [26–28]. In particular, a number of studies suggest that influential researchers and key opinion leaders, often identifiable through their citation counts (i.e. highly cited researchers (HCRs)), play a pivotal role in determining the direction of an emerging field by consolidating new paradigms and advancing the boundaries of existing knowledge [29–31]. These HCRs frequently occupy central positions within research networks, contributing more significantly to technological advancements and shaping the work of subsequent researchers due to their positions of influence [32,33]. Consequently, by identifying the HCRs in the PeSC field and analysing their lifetime research topics and surrounding research networks, we can gain deeper insights into critical issues in the evolution of PeSC research. These include the intellectual landscape of PeSC, knowledge exchanges between PeSC research and adjacent fields and the interplay among next-generation solar cell technologies.

In this study, we systematically investigated the evolution and thematic changes in PeSC research through comprehensive bibliometric analyses, including citation relationships, publication trends in PeSC-related fields, PeSC-related highly cited papers (HCPs) and keyword and collaboration networks. We also provided an in-depth analysis of researchers who have made significant contributions to the PeSC research community. Specifically, we identified researchers who published 10 or more HCPs between 2009 and 2021, designating them as PeSC-HCRs. We then examined their overall research activities from 2005 to 2022 using social network analysis (SNA) based on author keywords. To ensure effective and methodological research, we classified all publications according to their publication years and InCites citation topics categorization. This approach enabled us to discern shifts in research priorities and to observe the emergence of new subdisciplines over time. Finally, our investigation was extended to the evolving patterns of research collaboration among PeSC-HCRs to gain insight into the evolution, diversification and contribution of collaborative networks to the advancement of the PeSC field.

This study aimed to assess the impact of PeSCs on various research fields, the reciprocal effects of these fields on PeSCs development, researcher migration and future projections. This innovative analytical approach is the first of its kind, and the insights derived from this work are expected to serve as a foundational reference for forecasting the developmental trajectories of other emerging technologies in the future.

## 2. Methodology

### 2.1. Data collection

**Figure 1.**
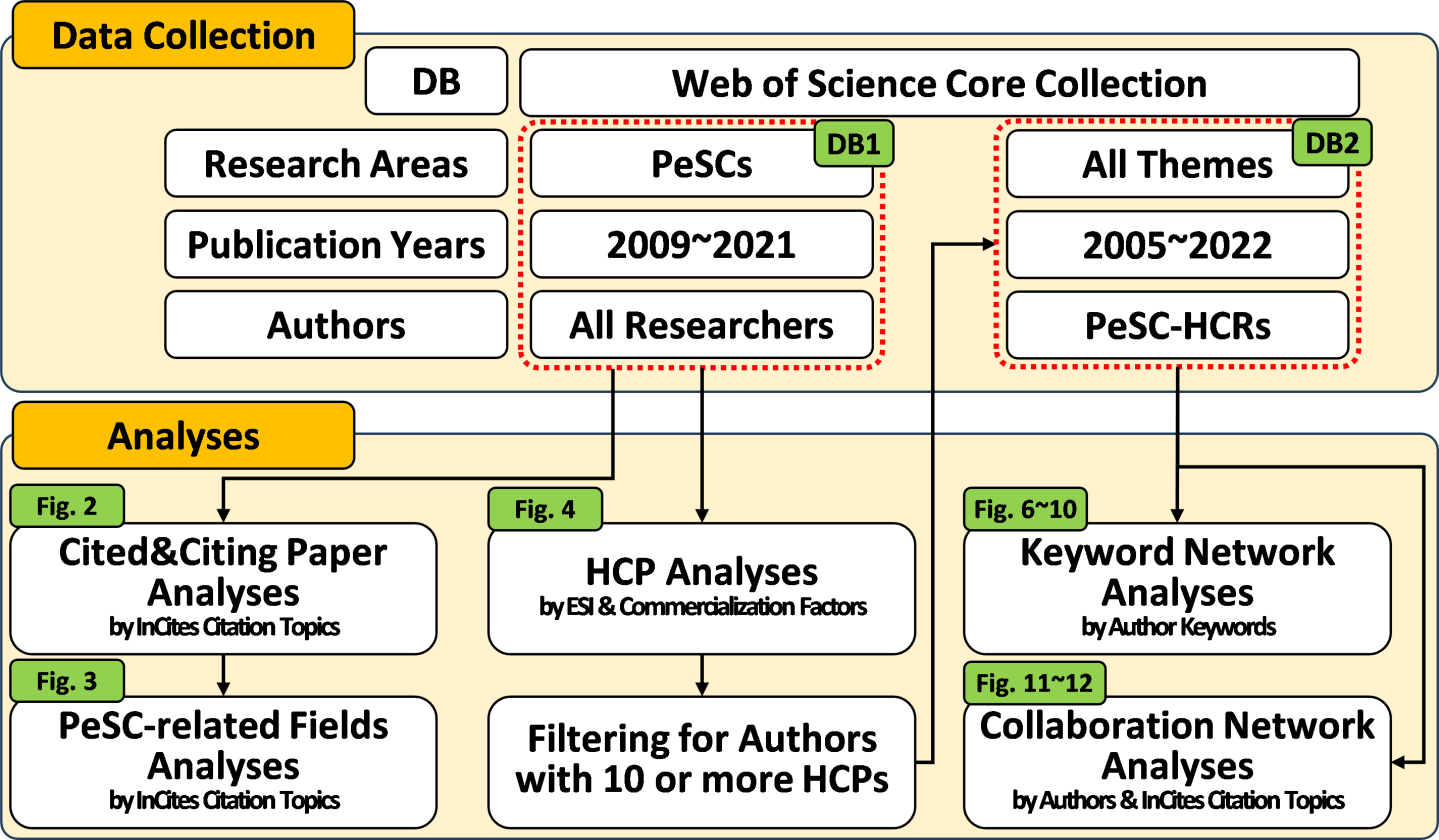
Schematic overview of the methodologies employed in this study, including figure numbers corresponding to the results.

All procedures are illustrated in figure 1. The data (DB1) were collected from the Web of Science Core Collection provided by Clarivate Analytics, with a search timespan set from 2009 to 2021. The index terms used for the search were ‘perovskite solar cell’ or ‘perovskite photovoltaic’. The initial search yielded 22 575 records. After expert reviews, 15 171 records were confirmed to be directly related to PeSCs. Once DB1 was finalized, we exported the necessary information for this study, including publication years, author keywords, abstracts, institutions, countries and cited references. The prepared DB1 was then uploaded to Web of Science and InCites Benchmarking & Analytics to retrieve information on citing papers, HCPs and InCites citation topics. The classification by InCites citation topics is generated through an algorithm that analyses citation relationships, providing an accurate reflection of the evolution of literature topics.

### 2.2. Citation relationship analysis

A total of 1836 cited references from PeSC papers published between 2009 and 2014 were extracted. Additionally, 31 806 papers that cited PeSC papers published between 2020 and 2022 were retrieved from the Web of Science system and DB1, as of 1 September 2023. These papers were analysed along with their InCites citation topics to explore the associated research areas. To focus on the influence of PeSCs on external fields, the PeSC topic was excluded when calculating the weight of articles by topic.

The results of the citation relationship analysis were used to identify the top 20 most-cited fields (InCites citation topics at the micro-level) related to PeSC papers. A trend analysis was then performed using InCites Benchmarking & Analytics to evaluate the annual number of publications in these areas.

### 2.3. Highly cited papers and perovskite solar cells highly cited researchers analysis

We utilized InCites Benchmarking & Analytics along with DB1 to analyse HCPs within the field of PeSCs (PeSC-HCPs). HCPs are defined as the top 1% of papers in their respective fields based on citation counts for the year of publication, as per Essential Science Indicators (ESI). The PeSC-HCPs were categorized into six ESI subjects, namely chemistry, engineering, environment/ecology, materials science, multidisciplinary and physics. To calculate the proportion of PeSC-HCPs relative to the total HCPs, we used the total number of HCPs assigned to each ESI subject as the denominator. In addition to classifying HCPs by ESI subjects, we categorized PeSC-HCPs based on commercialization factors such as efficiency, stability, public acceptability, scaling-up and cost [19].

We also identified researchers who published 10 or more HCPs between 2009 and 2021 and designated them as PeSC-HCRs. We then created another database (DB2) by searching for these 69 PeSC-HCRs in the Web of Science. To comprehensively explore their entire research trajectory, we set the search timespan from 2005 to 2022, which is broader than that used for DB1. This period was chosen because the number of publications prior to 2005 was extremely low, making it unlikely that earlier years would make a meaningful contribution to the analysis. This period also covers recent trends, including the peak publication year of 2021, followed by a decline in 2022. We followed the same procedures used for DB1, including the merging of synonyms into representative chemical symbols, words and abbreviations.

### 2.4. Social network analysis for keyword and collaboration networks

From DB2, author keywords were extracted as the primary data because they provide the most concise representation of the research papers. For the purpose of temporal analysis, the publication records were segmented into five periods based on publication years: 1359 records from 2005 to 2011, 1895 records from 2012 to 2014, 2981 records from 2015 to 2017, 2248 records from 2018 to 2019 and 3174 records from 2020 to 2022, totalling 11 657 records. To generate keyword and collaboration networks, the prepared database (DB2) was imported into NetMiner4 (Cyram), a bibliometric software tool designed for visual and interactive analysis of structured and unstructured data. The networks were constructed using the co-occurrence of author keywords and co-authorship data. Subsequently, betweenness centrality was calculated for each node, and cohesive communities within the network were analysed to form distinct groups through hierarchical clustering [34]. For keyword networks, group topics were determined based on the allocated keywords for each group. However, groups with fewer than three keywords were not assigned a group topic due to insufficient information. For collaboration networks, the analysis was divided into two periods—2005−2011 and 2020−2022—to contrast the collaboration patterns before and after the advent of PeSC research. Furthermore, the colour of each node represents the InCites citation topics of the respective researchers, providing insights into their specific areas of focus. InCites citation topics were designated for respective researchers based on the field in which they published the most papers in a given year. We also analysed individual node parameters, such as betweenness centrality and ego-network properties. An ego network consists of a single actor (ego), the actors they are connected to (alters) and the links among those alters [35]. Furthermore, network properties, such as density and external–internal (E–I) index, were examined. Network density was calculated as the ratio of the number of actual connections to the maximum possible connections in the network [36]. The E–I index compared the number of links connecting nodes outside the group to those within the group [19,25]. The index ranges from −1 to 1, where −1 indicates that all links are within the same group, and 1 indicates that all links connect to nodes outside the group.

## 3. Results and discussion

**Figure 2.**
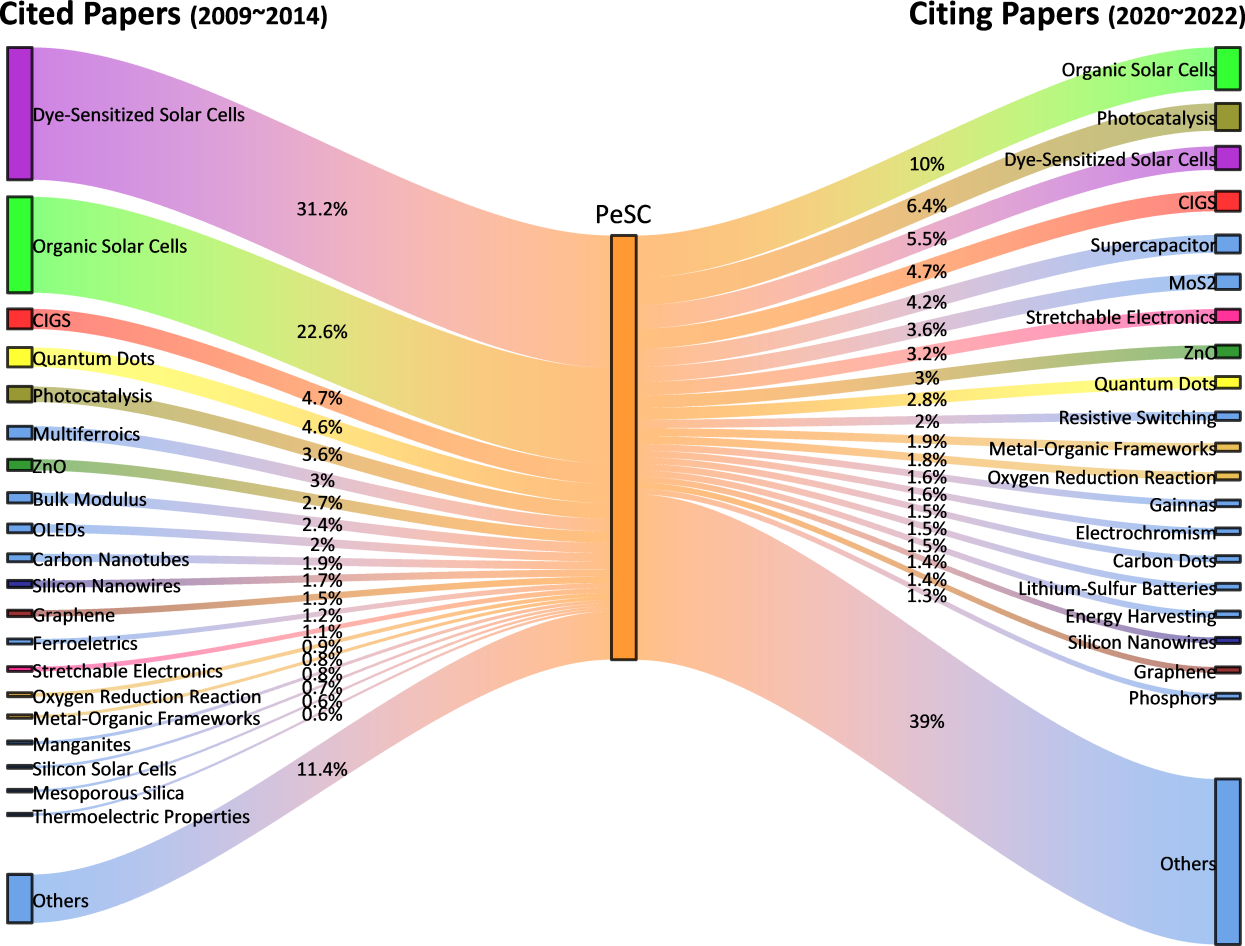
Relative proportions of research areas (InCites citation topics) cited by PeSC papers published during 2009−2014 and those citing PeSC papers published during 2020−2022. In the analysis of citing PeSC papers (right-hand), PeSC field itself was excluded to focus on the influence of PeSC research on external fields.

The citation relationships between PeSCs and other research fields were analysed by focusing on two distinct phases (figure 2): the initial phase from 2009 to 2014, representing the early stages of PeSC research, and a more mature phase from 2020 to 2022. For the research domain classification, we used the micro-level citation topic classification from the InCites database.

In the initial phase, the research topics of papers cited in PeSC publications were examined to understand the research areas that influenced the early development of PeSC technology. From 2009 to 2014, papers on DSSCs were the most frequently cited in PeSC publications, followed by OSCs, accounting for 31.2 and 22.6% of the total references, respectively. In addition to DSSCs and OSCs, research on various technological fields also contributed to the growth of the PeSC research community, though papers from these fields each accounted for less than 5% of all references. This suggests that early PeSC publications predominantly cited work related to next-generation PVs, indicating that research on DSSCs and OSCs probably played a pivotal role in stimulating the growth of PeSC technology. Contrarily, citing papers published in the mature phase (2020−2022) exhibited diverse distributions across various scientific domains such as photocatalysis, supercapacitors, stretchable electronics and ZnO. Thus, as PeSC technology evolved, there has been a notable impact on technological advancements in various fields involving the use of perovskite materials.

**Figure 3.**
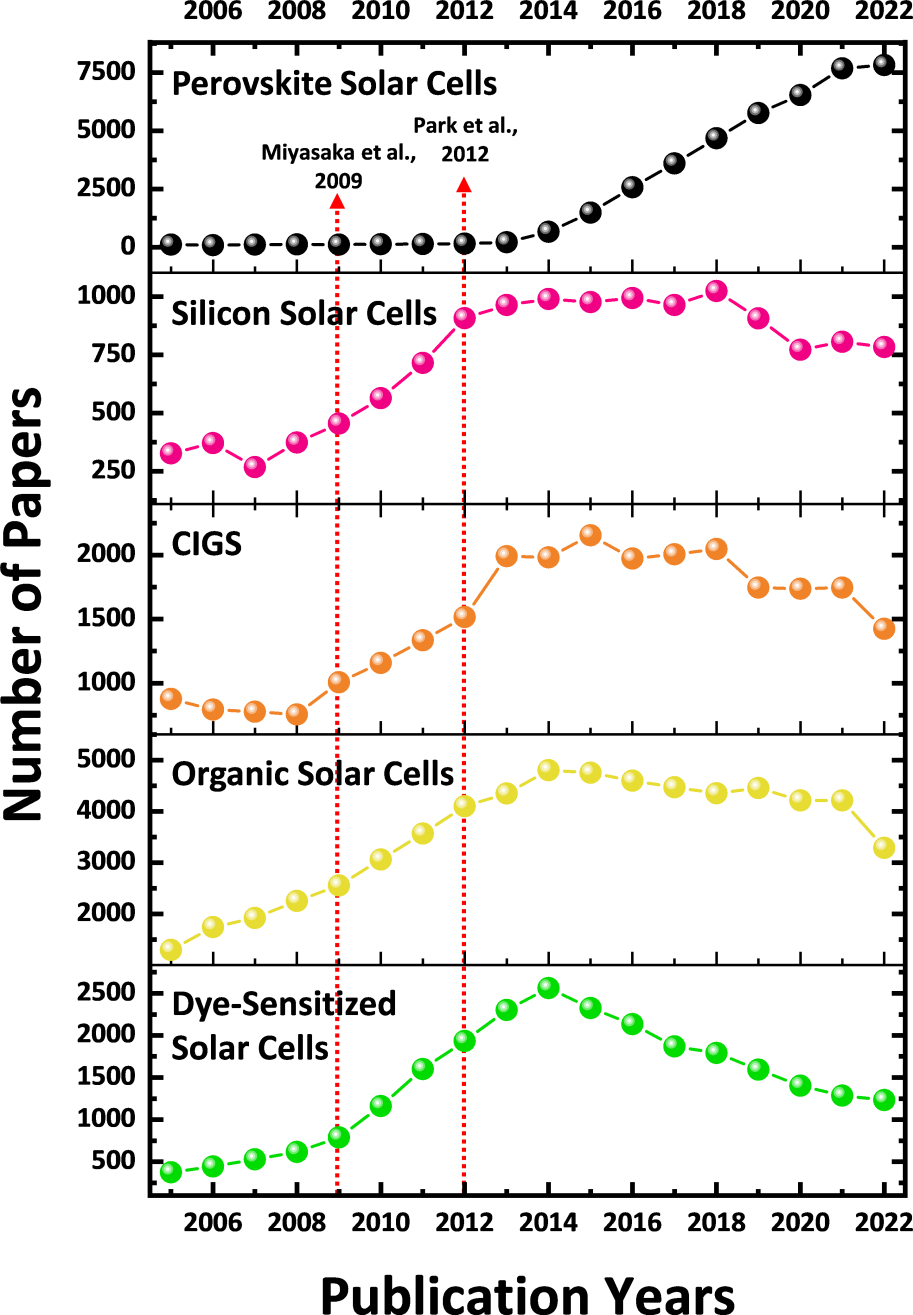
Annual publication trends in various PV research areas from 2005 to 2022.

Based on the citation relationship results, we conducted a trend analysis on the annual number of publications from 2005 to 2022 in the top 20 most-cited fields in PeSC papers, as shown in figure 3 and electronic supplementary material, figure S1. We compared the total number of publications on PeSCs, DSSCs and OSCs, alongside other PV technologies (figure 3). This approach enabled us to investigate the impact of the emergence of PeSCs on the publication volumes in other PV sectors. The most productive PV field, in terms of publication volume, varied over time. Prior to 2014, there was a predominant focus on next-generation PVs based on organic materials, such as OSCs and DSSCs, over their inorganic counterparts, including Si and copper indium gallium selenide (CIGS).

Since 2014, however, PeSC publications have skyrocketed, which can be attributed to several pioneering studies conducted in 2012 that demonstrated the feasibility of organic–inorganic hybrid perovskites as photoactive materials [8,37,38]. Conversely, CIGS, OSC and DSSC fields have experienced a noticeable decline in publication volume since their peak in 2014 and 2015, which is consistent with other research results [12,39]. These findings suggest that research priorities within the PV community have progressively shifted towards the PeSC domain since 2014. Notably, the annual number of publications related to Si solar cells did not show a noticeable correlation with the rise in PeSC papers, indicating that PeSC research was not directly influenced by Si solar cell technology.

Although basic statistics provide a valuable overview of trends in various fields surrounding PeSC technology, fully elucidating the changes in the PeSC research landscape based solely on these numbers remains challenging. To address this gap, we incorporated an in-depth bibliometric method combined with HCP and PeSC-HCR analyses. HCPs represent the top 1% of papers in their respective ESI subjects per year, serving as a proxy for expertise and research trends within the field. Through HCP analysis, PeSC-HCRs—researchers with 10 or more HCPs during the period 2009−2021—were identified. The advantages of the PeSC-HCR-based approach are as follows: [30–32] (i) the scientific trajectory of HCRs reveals the areas they have influenced in the past and the current research directions; (ii) this allows the analysis of a wide range of fields, not limited to the PeSC; and (iii) it also allows the examination of interconnectivity and interaction dynamics across various fields, with a particular focus on PeSC research.

**Figure 4.**
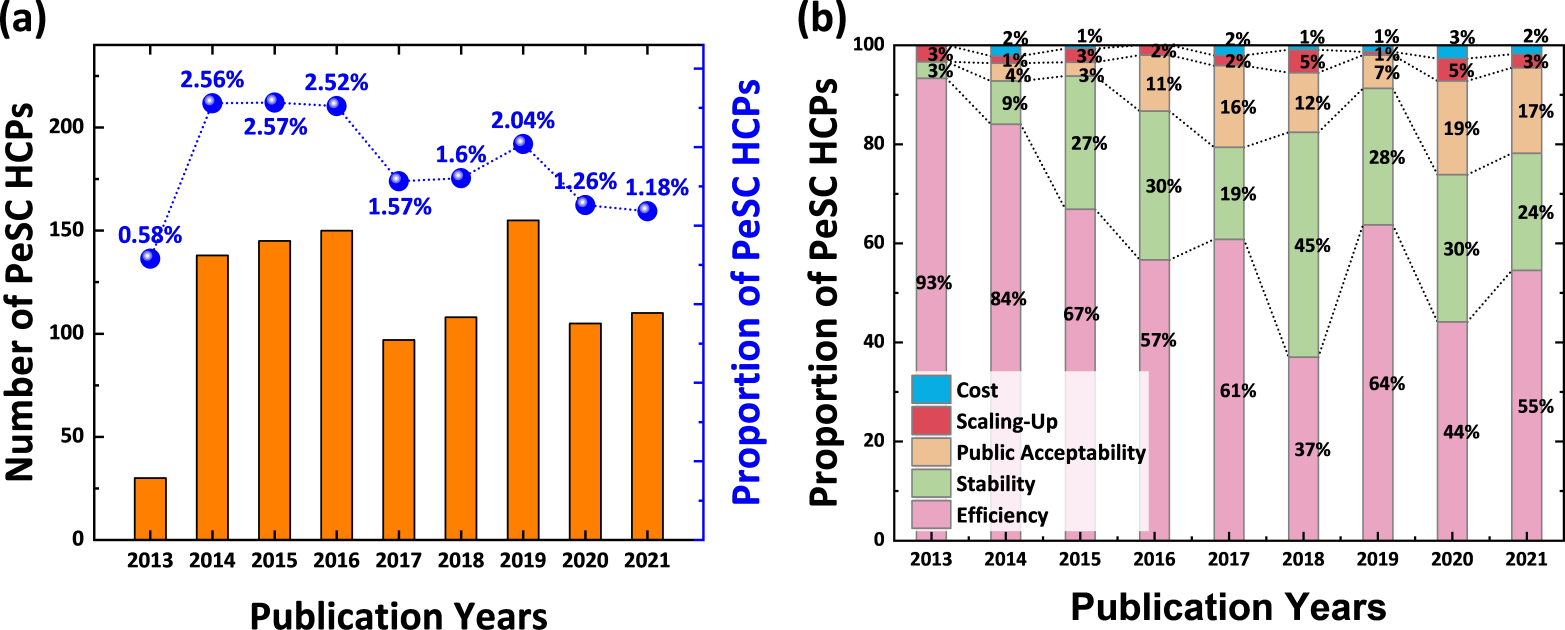
(a) The number of PeSC-HCPs published from 2013 to 2021 and their relative proportions, and (b) the distribution of PeSC-HCPs according to commercialization factors.

Figure 4a shows the temporal trends in the volume of PeSC-HCPs and their relative proportions across all fields of HCPs. Although no consistent pattern emerged over the years, PeSC-HCPs accounted for more than 2.5% of all HCPs during the period 2014−2016, indicating a significant impact of PeSCs research on the broader scientific community. However, since 2017, a declining trend in both volume and proportion has been observed; this downward trend was particularly pronounced in 2021 when PeSC-HCPs represented only 1.18% of all HCPs. This tendency may reflect a fundamental shift in the structural evolution of the field. As citations often concentrate around early seminal works, more recent studies—typically more specialized and incremental—tend to receive fewer citations. As a result, a natural tapering in HCP representation can be observed over time.

In addition to these trends, the distribution of HCPs for each ESI subject changed, as listed in electronic supplementary material, tables S1 and S2. A comparison between 2016 and 2019, with similar numbers of HCPs, showed an increase in HCPs in subjects related to applied science subjects, such as engineering and material science, alongside a noticeable decrease in HCPs in basic science subjects, including physics and chemistry. This can be interpreted as the technological progress of PeSCs and their entry into the maturation and implementation phases.

To identify which sub-areas within PeSC research generated the most HCPs, PeSC-HCPs were also classified according to their commercialization factors, such as efficiency, stability, public acceptability, scaling-up and cost. In the initial phase of the study, HCPs related to efficiency accounted for approximately 90% of the total (figure 4b). However, in recent years, the proportion of HCPs focused on efficiency has noticeably decreased, while the proportions related to stability and public acceptability have significantly increased. Specifically, the negligible share in 2013 increased to 24 and 17% in 2021 for stability and public acceptability, respectively. These HCP-based results agree with the trends observed in the analysis of all publications [19]. Meanwhile, the proportions of HCPs related to costs and scaling-up remained below 5% throughout all periods.

**Figure 5.**
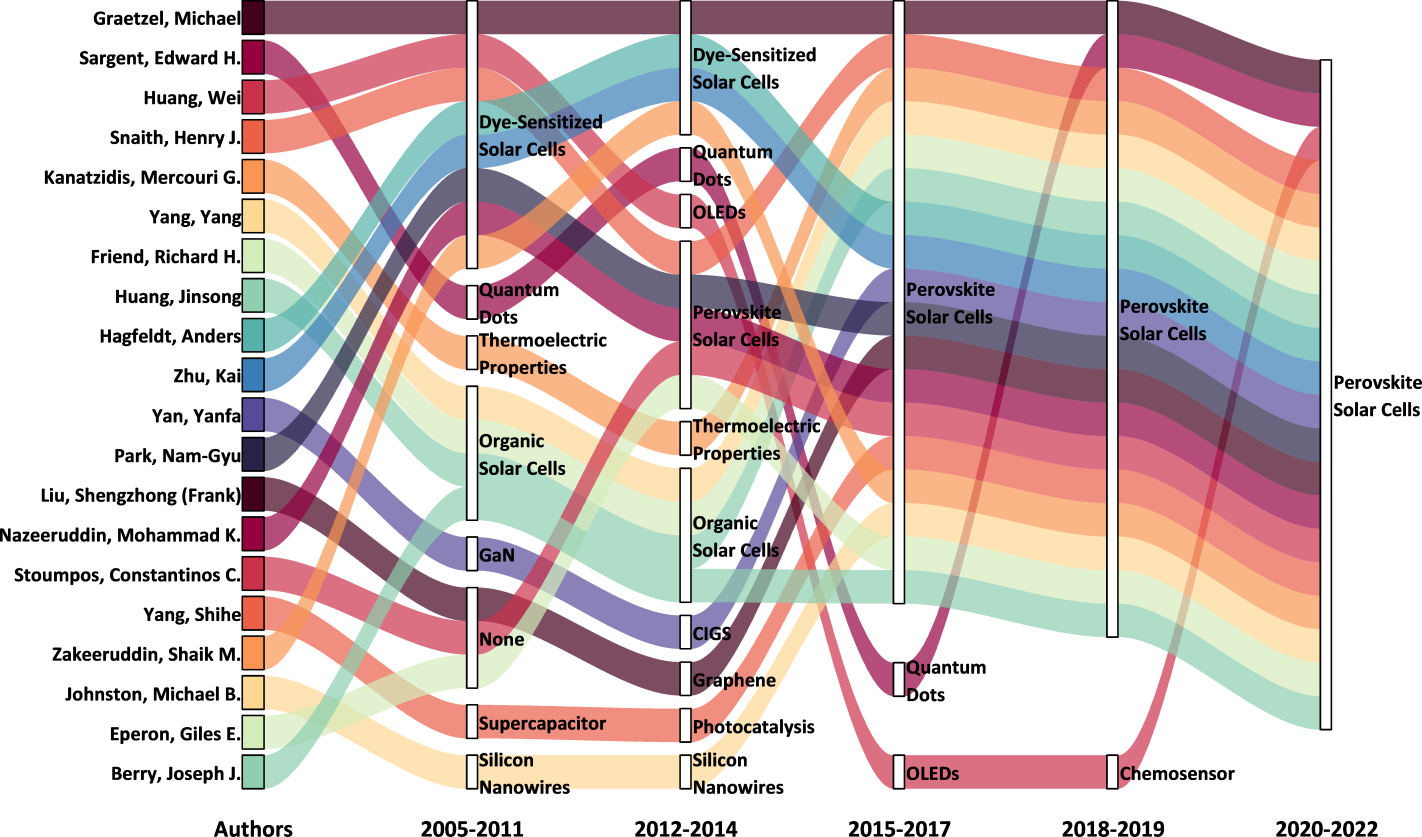
Changes in research focus (InCites citation topics) of the top 20 PeSC-HCRs from 2005 to 2022.

To gain more comprehensive insights into the knowledge structures within the PeSC domains, we identified 69 researchers who published 10 or more HCPs between 2012 and 2021. Given the large volume of publications and the broad community of researchers in the PeSC field, focusing our analysis on this select group of HCRs allowed for a more targeted and detailed review of advancements in PeSC research. We analysed all publications from 2005 to 2022 by dividing the period into five different intervals: 2005−2011, 2012−2014, 2015−2017, 2018−2019 and 2020−2022. Figure 5 depicts the temporal evolution of the research themes of the top 20 PeSC-HCRs, illustrating the shifts in their research focus. In the pre-PeSC era (2005−2011), PeSC-HCRs were engaged in various fields, including DSSCs, quantum dots (QDs), thermoelectric devices, OSCs, GaN, supercapacitors and silicon nanowires. These flows demonstrate that most PeSC-HCRs were influential researchers from other fields who later transitioned into PeSC research rather than researchers who initially entered the PeSC field directly. PeSC-HCRs associated with DSSCs, including Henry J. Snaith, Nam-Gyu Park and Mohammad K. Nazeeruddin, have changed their main research focus to PeSCs more quickly than to OSCs. In contrast, researchers, such as Yang Yang and Richard H. Friend, who were originally focused on the OSC field, changed their main research area to PeSCs at a relatively later stage. This trend is evidenced by early PeSC studies published in 2012, which universally adopted mesoscopic negative-intrinsic-positive (n-i-p) architectures derived from DSSC designs [8,37,38]. This evolution can be traced back to the first reported PeSC by Kojima *et al.* [5], which introduced a perovskite-sensitized DSSC configuration, utilizing a mesoporous TiO_2_ scaffold and a liquid electrolyte. Although this work provided crucial proof of concept, limitations in efficiency and stability led to a series of follow-up studies—particularly in 2012—that replaced liquid components with solid-state hole transport materials and further refined the DSSC-based framework. These developments were largely led by researchers from the DSSC community, which helps explain their early prominence in shaping the PeSC field.

In addition, several PeSC-HCRs, such as Giles E. Eperon and Constantinos C. Stoumpos, were not associated with any designated research field during the pre-PeSC period (2005−2011), probably indicating that they were early-career researchers at the time. These researchers entered the PeSC field between 2012 and 2014 and collaborated with established PeSC-HCRs during their early contributions to the field: Giles E. Eperon collaborated with Henry J. Snaith [1,40], and Constantinos C. Stoumpos collaborated with Mercouri G. Kanatzidis [41]. While these collaborations with influential researchers may have played a role in their entry into PeSC research, it is possible that their individual research capabilities also contributed to their success.

Table 1 presents the ranking of the InCites citation topics covered by all PeSC-HCRs. DSSCs, OSCs and organic LEDs (OLEDs) consistently maintained high rankings throughout the study period. However, there was a significant drop in the ranking, particularly for DSSCs, starting in the 2018−2019 period. More recently, research on thermoelectric properties, CO_2_ reduction and metal–organic frameworks has gained prominence.

**Table 1.** Ranking of research fields (InCites citation topics) according to the number of papers published in the respective periods.

	2005−2011	2012−2014	2015−2017	2018−2019	2020−2022
**1**	dye-sensitized solar cells	dye-sensitized solar cells	organic solar cells	organic solar cells	organic solar cells
**2**	organic solar cells	organic solar cells	dye-sensitized solar cells	OLEDs	OLEDs
**3**	OLEDs	OLEDs	OLEDs	chemosensor	thermoelectric properties
**4**	ZnO	photocatalysis	photocatalysis	dye-sensitized solar cells	CO_2_ reduction
**5**	quantum dots	standard model	quantum dots	thermoelectric properties	metal–organic frameworks
**6**	photocatalysis	CIGS	metal–organic frameworks	metal–organic frameworks	CIGS
**7**	thermoelectric properties	quantum dots	chemosensor	supercapacitor	quantum dots
**8**	SbSI	ZnO	CIGS	CIGS	supercapacitor
**9**	self-assembled monolayers	thermoelectric properties	thermoelectric properties	MoS_2_	oxygen reduction reaction
**10**	chemosensor	chemosensor	supercapacitor	CO_2_ reduction	photocatalysis
**11**	carbon nanotubes	supercapacitor	MoS_2_	oxygen reduction reaction	chemosensor
**12**	CIGS	graphene	oxygen reduction reaction	quantum dots	resistive switching
**13**	graphene	SbSI	ZnO	magnetic nanoparticles	dye-sensitized solar cells
**14**	resistive switching	self-assembled monolayers	phosphors	photocatalysis	deep learning
**15**	GaAs	metal–organic frameworks	graphene	iron-based superconductors	SbSI
**16**	electromigration	silicon nanowires	silicon nanowires	self-assembled monolayers	stretchable electronics
**17**	single-molecule magnets	resistive switching	silicon solar cells	stretchable electronics	magnetic nanoparticles
**18**	hyperpolarizability	hyperpolarizability	aptamer	deep learning	iron-based superconductors
**19**	Kondo effect	silicon solar cells	self-assembled monolayers	silicon solar cells	coronavirus
**20**	porous silicon	GaAs	iron-based superconductors	ZnO	bulk modulus

**Figure 6.**
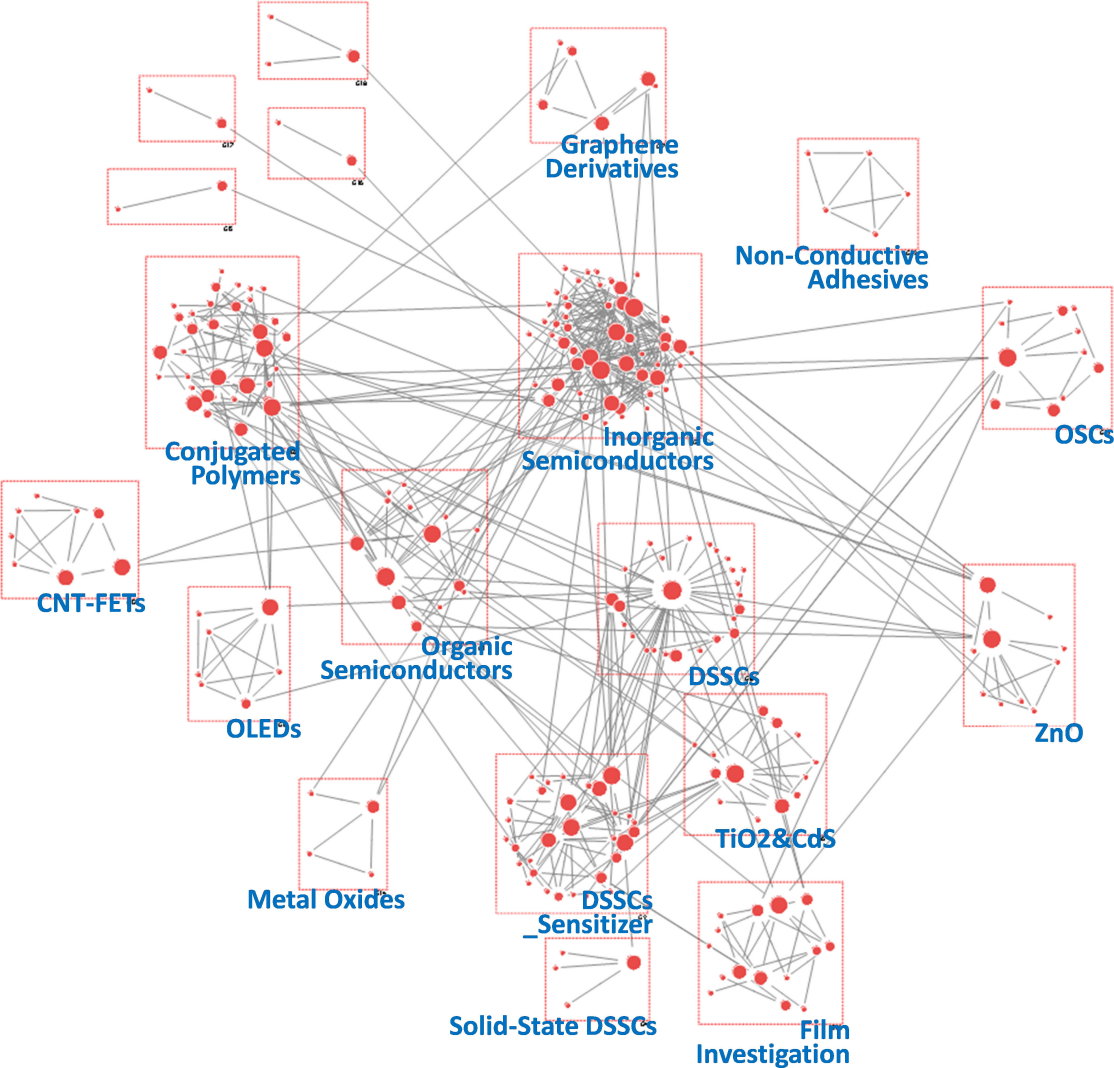
Keyword network derived from author keywords in papers published during the period 2005−2011. CNT-FET: carbon nanotube field-effect transistor.

**Figure 7.**
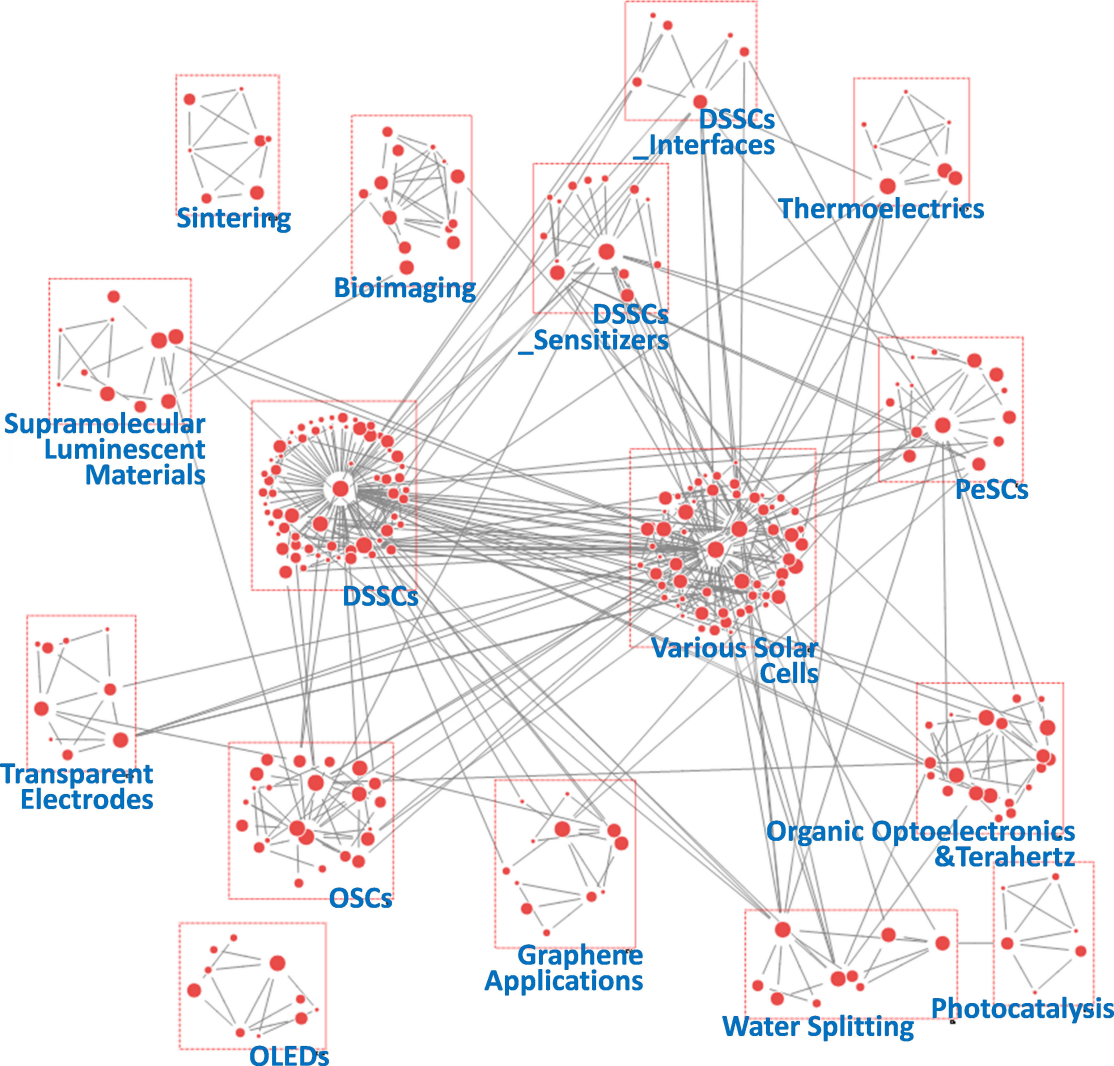
Keyword network derived from author keywords in papers published during the period 2012−2014.

**Figure 8.**
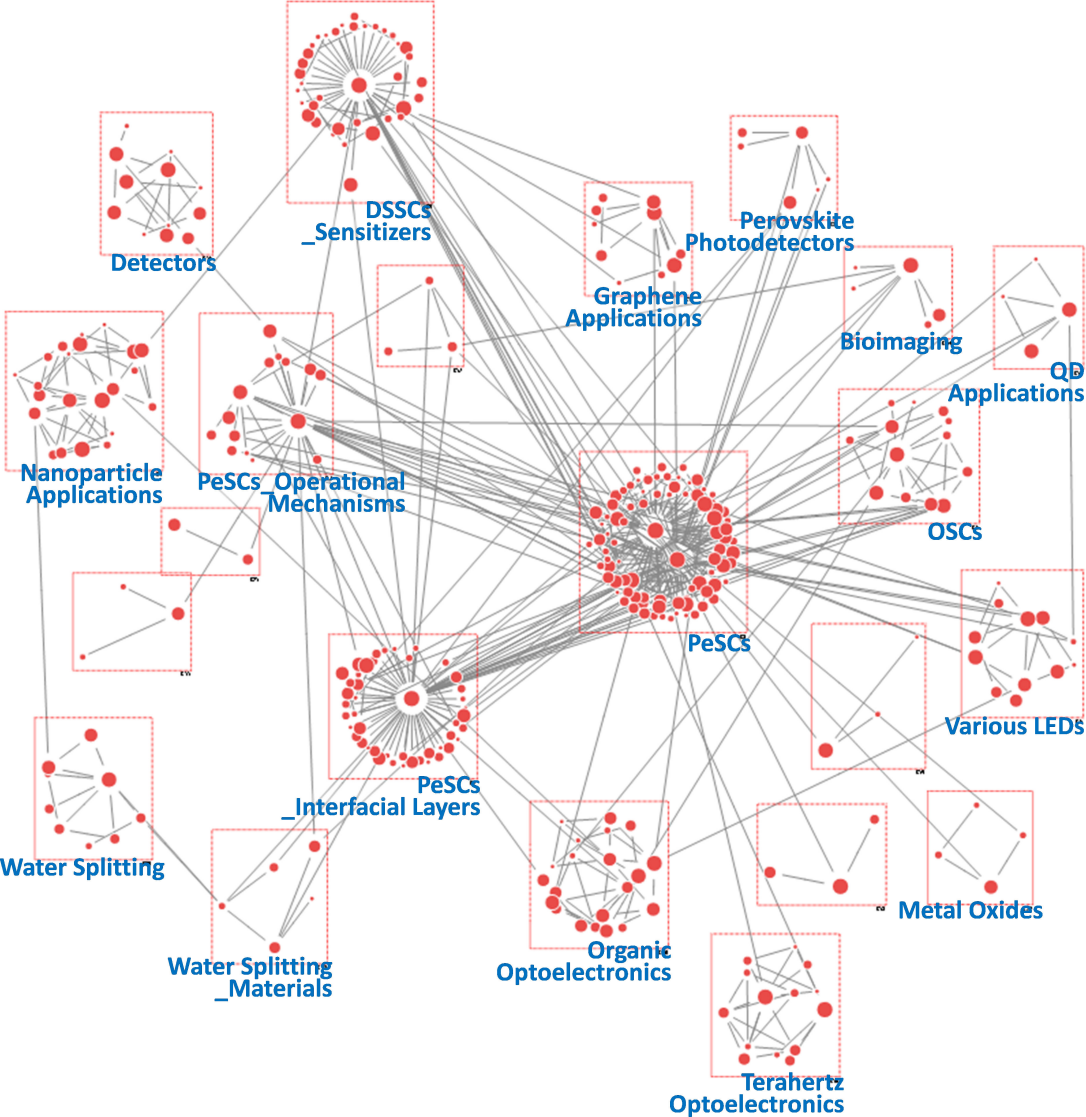
Keyword network derived from author keywords in papers published during the period 2015−2017.

**Figure 9.**
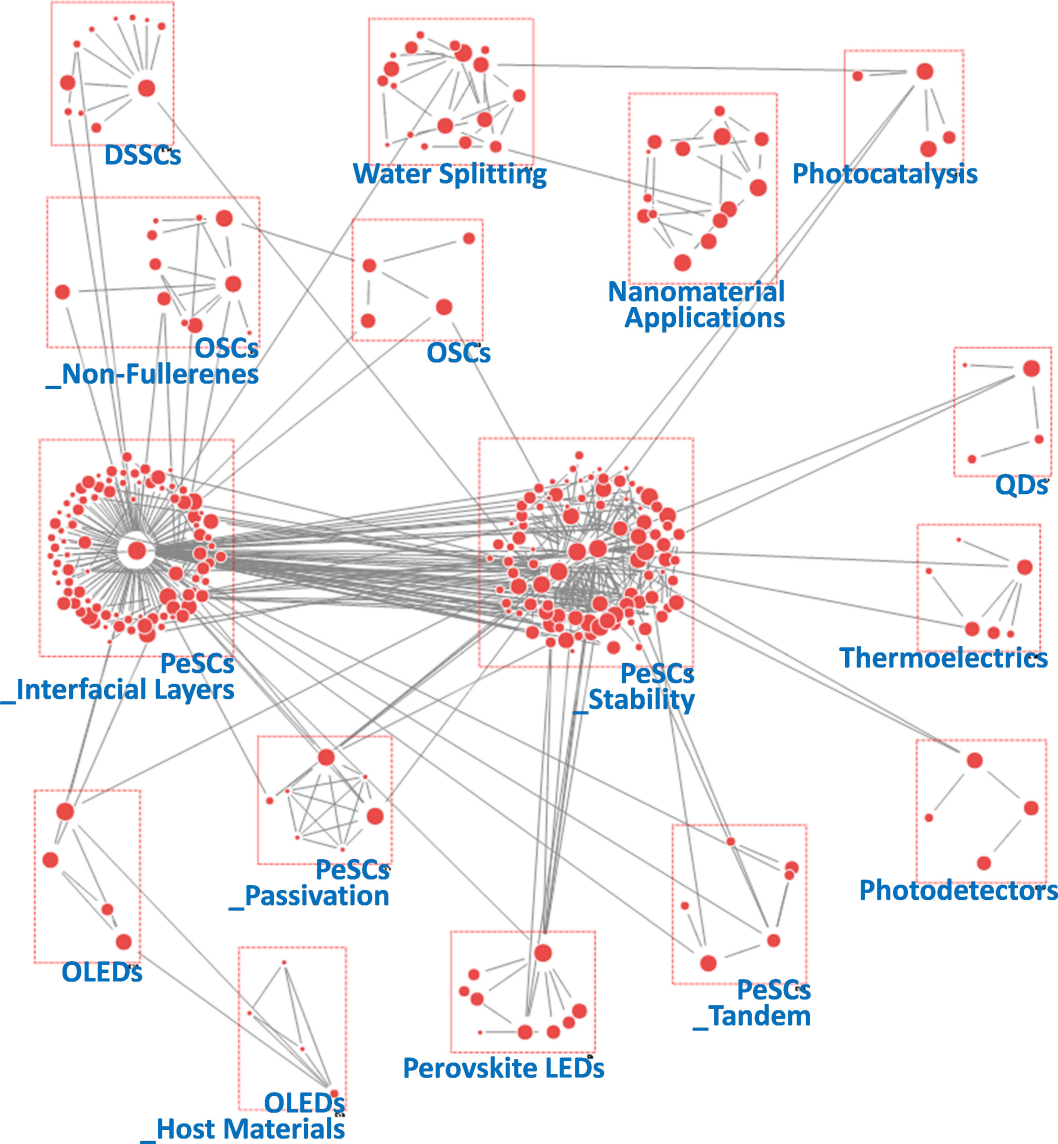
Keyword network derived from author keywords in papers published during the period 2018−2019.

**Figure 10.**
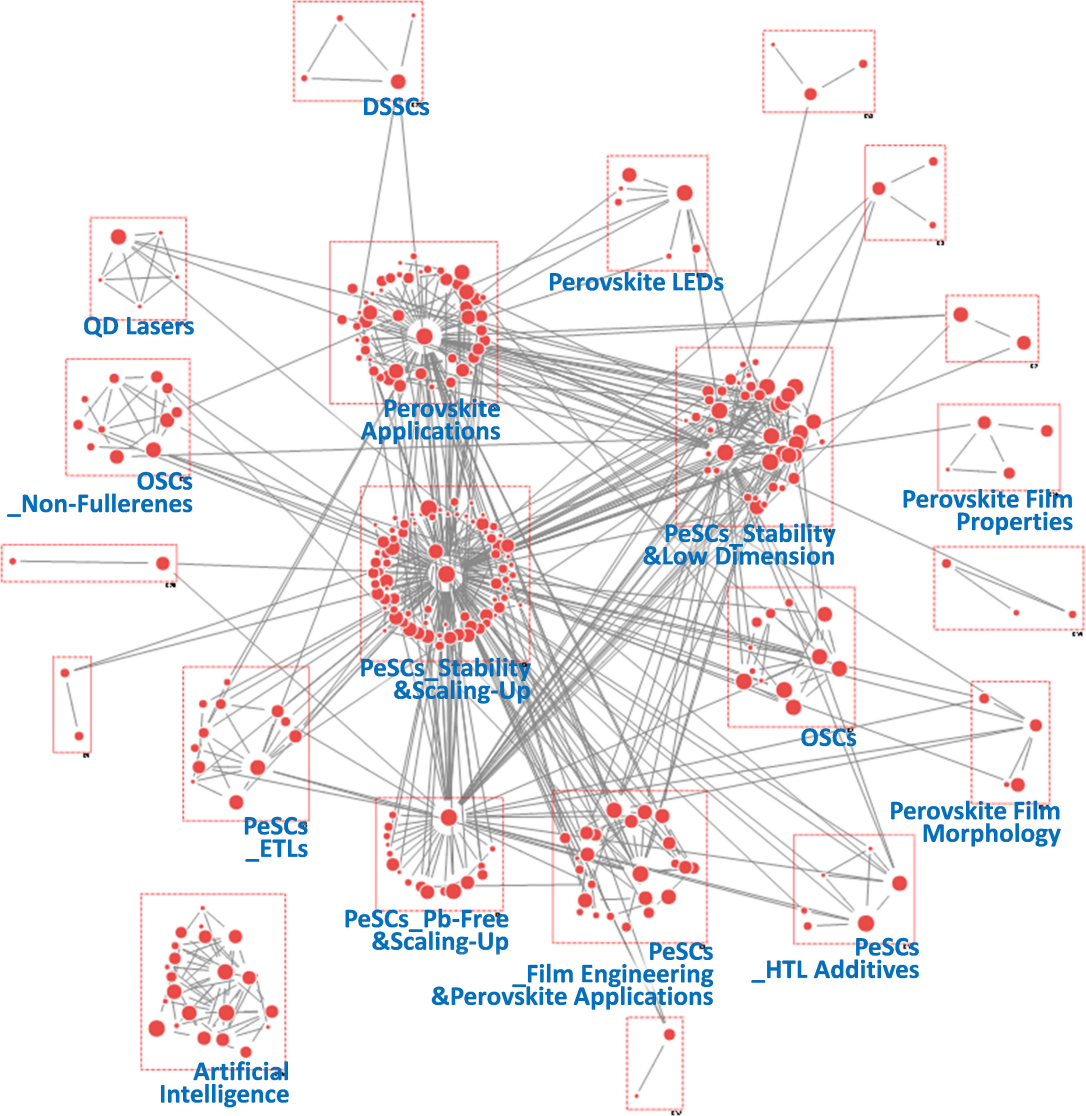
Keyword network derived from author keywords in papers published during the period 2020−2022.

To obtain more detailed insights into research dynamics, we conducted SNA using author keywords from all PeSC-HCR publications. The nodes in the network were derived from author keywords, and the links in the network were based on the co-occurrence of these keywords. Unlike co-citation analysis, which is concerned with references, co-word analysis examines the content of publications directly, with the term or ‘word’ as the fundamental unit of analysis [36]. This technique operates under the assumption that words that frequently co-occur within a text are thematically related. We employed hierarchical clustering to group these thematically related words into distinct clusters [34], enabling us to identify key themes and patterns within the research domain. This approach provides a relational and visual representation of the knowledge structure, making it particularly useful for illustrating how certain terms cluster together and how various research areas are interconnected [25,42]. Additionally, we adopted betweenness centrality in our keyword network analysis. This metric measures how often a node acts as a bridge on the shortest path between two other nodes, thereby identifying key information channels and interdisciplinary links [19,36]. Figures 6–10 present the resultant keyword networks along with the identified group topics. Specific information on the composite keywords is listed in electronic supplementary material, tables S3–S7.

A total of 19 groups were identified for the period 2005−2011 (figure 6). The largest group was ‘Inorganic Semiconductors’, which contained 52 keywords and was connected to various other groups, including ‘Organic Semiconductors’, ‘Conjugated Polymers’, ‘DSSCs’ and ‘OSCs’. Within the ‘Inorganic Semiconductors’ group, most keywords were related to the characteristics and fundamental principles of semiconductors, including ‘Density Functional Theory (DFT)’, ‘Photoluminescence (PL)’, ‘II-VI semiconductor’, ‘crystal structure’ and ‘energy gap’. In addition, three groups directly related to DSSCs were identified: ‘DSSCs’, ‘DSSCs_Sensitizer’ and ‘Solid-State DSSCs’. The keywords in these groups were linked to light absorbers used in DSSCs, such as ‘organic dye’, ‘quantum dot’, ‘sensitizer’, ‘squaraine’ and ‘PbS’. This suggests that researchers in the DSSC domain focused on improving light absorber performance and exploring innovative alternatives to overcome the limitations of conventional materials [43,44]. In the ‘Conjugated Polymers’ and ‘Organic Semiconductors’ groups, keywords related to OLEDs, such as ‘photophysics’, ‘polyfluorene’, ‘luminescence’ and ‘host-guest systems’, were frequently observed [45]. Additionally, groups associated with graphene, carbon nanotubes and metal oxides were also prominent [46,47]. As observed from the pre-PeSC era, various optoelectronic materials, including organic and inorganic semiconductors, have been intensively studied. This lays the foundation for hybrid organic–inorganic PeSCs.

Figure 7 shows the keyword network for the period 2012−2014, identifying 16 groups. Compared with the previous period, most groups were related to application-oriented topics with a focus on solar cell technologies such as ‘Various Solar Cells’, ‘DSSCs’ and ‘OSCs’. Moreover, the emergence of the ‘PeSCs’ group marks the beginning of PeSC research. Within the ‘Various Solar Cell’ group, numerous keywords related to photoactive materials appeared: ‘dye’, ‘CdTe’, ‘Si’, ‘CIGS’ and ‘copper zinc tin sulfide (CZTS)’. Notably, the groups related to DSSCs were divided into three categories: ‘DSSCs’, ‘DSSCs_Sensitizers’ and ‘DSSCs_Interfaces’. Keywords in these groups, such as ‘colloidal quantum dot’, ‘black dye’, ‘electrolyte’ and ‘charge transport’, suggest that researchers in the DSSC domain were focused on improving the performance of photo-sensitizers and optimizing the interfaces between photo-anodes and electrolytes [48,49]. In addition to PV technologies, diverse research topics were considered: ‘Thermoelectrics’, ‘Organic Optoelectronics&Terahertz’, ‘Water Splitting’ and ‘Bioimaging’ [50–52]. This broad spectrum of research domains implies that, before the concentrated focus on PeSCs, PeSC-HCRs were involved in various fields. This interdisciplinary foundation probably played an important role in the development of PeSC technology.

In the 2015−2017 period, 22 groups were observed and are depicted in figure 8. Among these, the number of DSSC-related groups decreased from three to one, while three sizeable PeSC-related groups emerged. The group of ‘PeSCs’ was connected to most groups within the network, linked by critical terms such as ‘stability’, ‘solution-processing’, ‘interface’ and ‘photochemistry’. These results suggest a pivotal transition in the research focus of PeSC-HCRs towards PeSCs. Notably, the ‘PeSCs_Interfacial Layers’ group includes keywords such as ‘fullerene’, ‘tin oxide’ and ‘reduced graphene oxide’, which were connected to the ‘OSCs’ group rather than the ‘DSSCs’ group [53,54]. This highlights the need for an interdisciplinary approach, merging insights from OSC research with those from PeSC research, particularly in the area of interfacial engineering [55]. The ‘PeSC_Operational Mechanisms’ group, focusing on keywords such as ‘hysteresis’, ‘perovskite phase’ and ‘recombination’, reflects a concerted effort by researchers to better understand and improve device performance [56]. For instance, a combined computational and experimental study demonstrated that the hysteresis observed in current–voltage curves mainly originates from low-moving ions [57]. Additionally, organohalide perovskites with low exciton binding energy reduce recombination rates, thereby improving charge transport [58,59]. This period also marked the beginning of research into other applications of perovskite materials. The connection between the water-splitting and PeSC groups was strengthened, and the emergence of ‘Perovskite Photodetectors’ group was observed. These phenomena highlight the growing recognition of the versatile potential of perovskite materials, beyond PV applications [60].

Figure 9 presents the keyword network for the period 2018−2019, identifying 16 groups, four of which were directly related to PeSCs. PeSC topics were further subdivided into stability, interfacial layers, tandem and passivation. This reflects a significant convergence of PeSC-HCRs towards PeSC-focused research during this period, aligning with the findings shown in figure 5. Notably, the ‘PeSC_Stability’ group emerged as the largest, highlighting the focused efforts of PeSC-HCRs to enhance the long-term reliability of PeSCs, which is considered a critical challenge for the commercial viability and widespread adoption of PeSC technology. Keywords in this group, such as ‘2D’, ‘inorganic perovskite’, ‘grain boundary’ and ‘interface engineering’, indicate that multi-faceted approaches were being employed, focusing active materials and chemical composition, defect passivation and interfacial engineering [61–63]. Importantly, this period also marked the first appearance of the perovskite LED group, signalling the expansion of perovskite material applications into LEDs. Additionally, the ‘PeSC_Stability’ and ‘Perovskite LEDs’ groups were connected through the keywords ‘quantum dot’ and ‘2D’. This connection suggests that perovskite QDs and 2D perovskites were being explored by the PeSC-HCR community as promising solutions for enhancing PeSC stability and replacing conventional light-emitting materials [64]. The ‘PeSCs_Interfacial Layers’ still showed a high degree of association with OSC-related groups, particularly ‘OSCs_Non-Fullerenes’. This strong link indicates that the exploration of fullerene alternatives is a key issue for both applications, and it may be possible to conduct simultaneous research on PeSCs and OSCs in terms of charge-transfer efficiency, interfacial stability and solution processability [65,66].

During the 2020−2022 period, 22 topic groups were identified (figure 10). Most groups were associated with PeSCs, and their specific topics were diversified, such as stability, low-dimensional structures, Pb-free materials, scaling-up, film engineering, hole transfer layer (HTL) additives and electron transfer layer (ETL). Owing to achievements in device efficiency and technology maturation, PeSC-HCRs have begun to focus on other challenges in commercializing PeSC technology, including stability, scaling-up and Pb-free issues. To address these challenges, inorganic cations, such as Cs^+^, have been investigated as substitutes for organic cations, such as methylammonium [67]. In addition, less toxic ions, such as Sn^2+^, Bi^3+^, Ge^2+^ and Mn^2+^, were investigated as potential alternatives to Pb^2+^ in perovskites [68]. Various strategies were developed for scaling-up, including roll-to-roll methods, encapsulation and module technologies [69]. Notably, PeSC-related groups covered multiple topics, suggesting an increasing complexity and interdisciplinary nature of advanced technology. For instance, ‘PeSC_Stability&Scaling-Up’ group included keywords related to both stability and scalability as follows: ‘scalability’, ‘thermal stability’, ‘inkjet printing’, ‘cross-linking’ and ‘encapsulation’. Furthermore, ‘PeSCs_ETLs’ and ‘PeSCs_HTL Additives’ groups included stability-related keywords referring to inorganic ETLs and new types of HTL dopants, respectively. Meanwhile, research on perovskite film engineering, represented by the ‘PeSCs_film Engineering&Perovskite Applications’ group, expanded into applications such as ‘resistive switching’, ‘memristor’ and ‘piezoelectric’, intermediated by 2D perovskite [70,71]. Furthermore, in ‘Perovskite Applications’, keywords, such as ‘X-ray imaging’, ‘X-ray detector’ and ‘scintillator’, demonstrated the potential of organometallic perovskites as promising scintillators, originating from their low-cost, high luminescence efficiency and tunable properties [21,72]. These research directions notably differ from earlier periods, when PeSC-HCRs primarily focused on perovskite LEDs. In the case of the DSSC, the number of groups and configuration nodes was significantly diminished, while the OSC maintained a certain group size, which is consistent with the results observed in table 1. An artificial intelligence (AI)-related group also emerged during this period. The keywords in this group collectively represent a comprehensive overview of these research trends in AI, with a strong emphasis on neural network architectures, data processing techniques and the application of AI in visual and predictive tasks [73]. This can accelerate research on new perovskite materials and compositions, device architectures and fabrication protocols optimization and device analyses.

In the final stage, we analysed the collaboration dynamics among PeSC-HCRs based on the SNA methodology, which provides a two-dimensional representation of the collaborative interactions and knowledge flow among the entities [25,42]. This approach visualizes collaborative patterns and quantifies the importance of specific nodes (or groups) through metrics such as degree, betweenness and closeness centralities. More importantly, a distinctive aspect of our analysis was the use of colour coding to represent the research fields of PeSC-HCRs according to their InCites citation topics. This study offers deep insight into interdisciplinary connections and illustrates the evolution and distribution of research interests at the researcher level.

**Figure 11.**
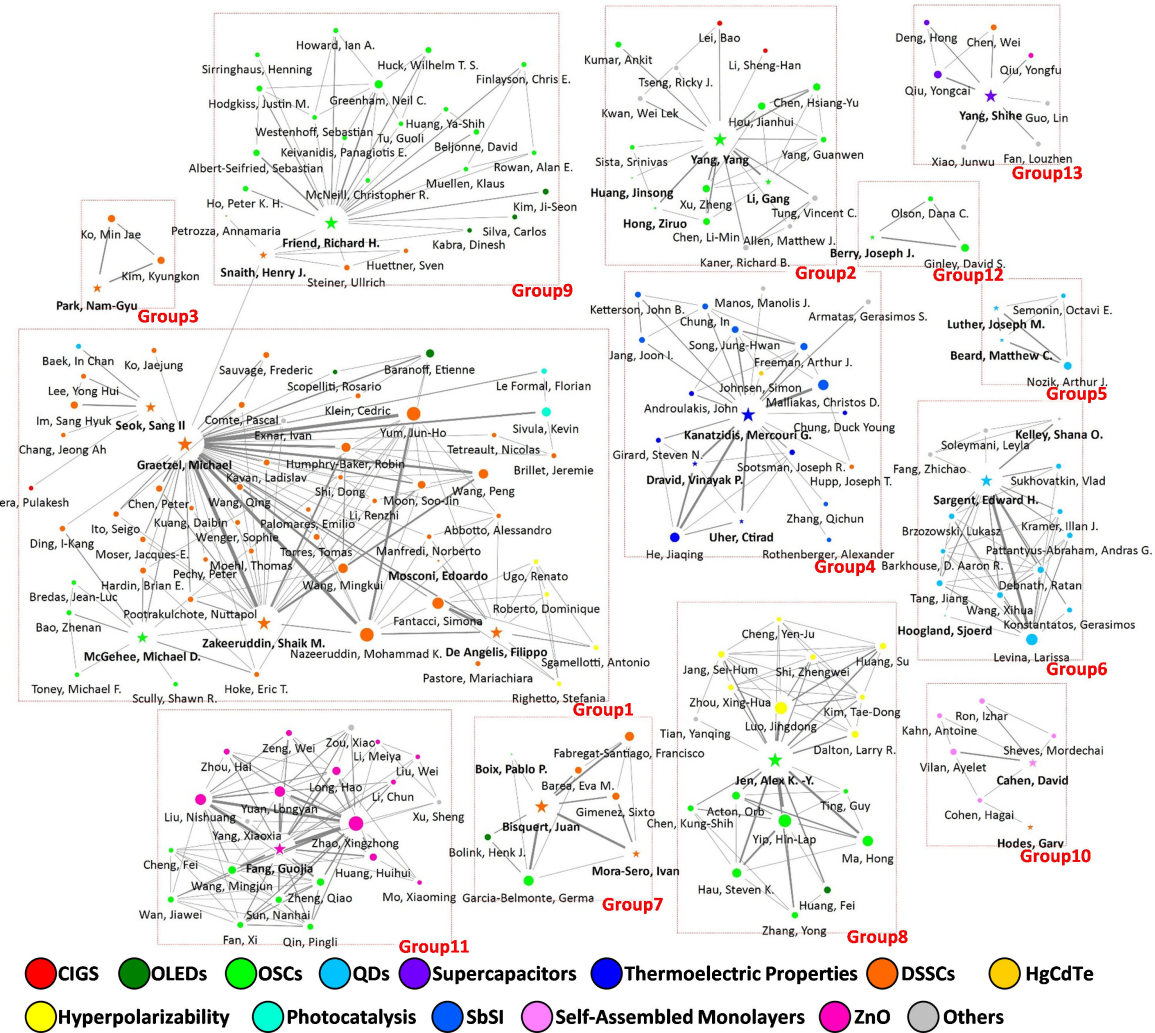
Collaboration network of researchers based on papers published during the period 2005−2011 with node colours representing respective InCites citation topics.

**Figure 12.**
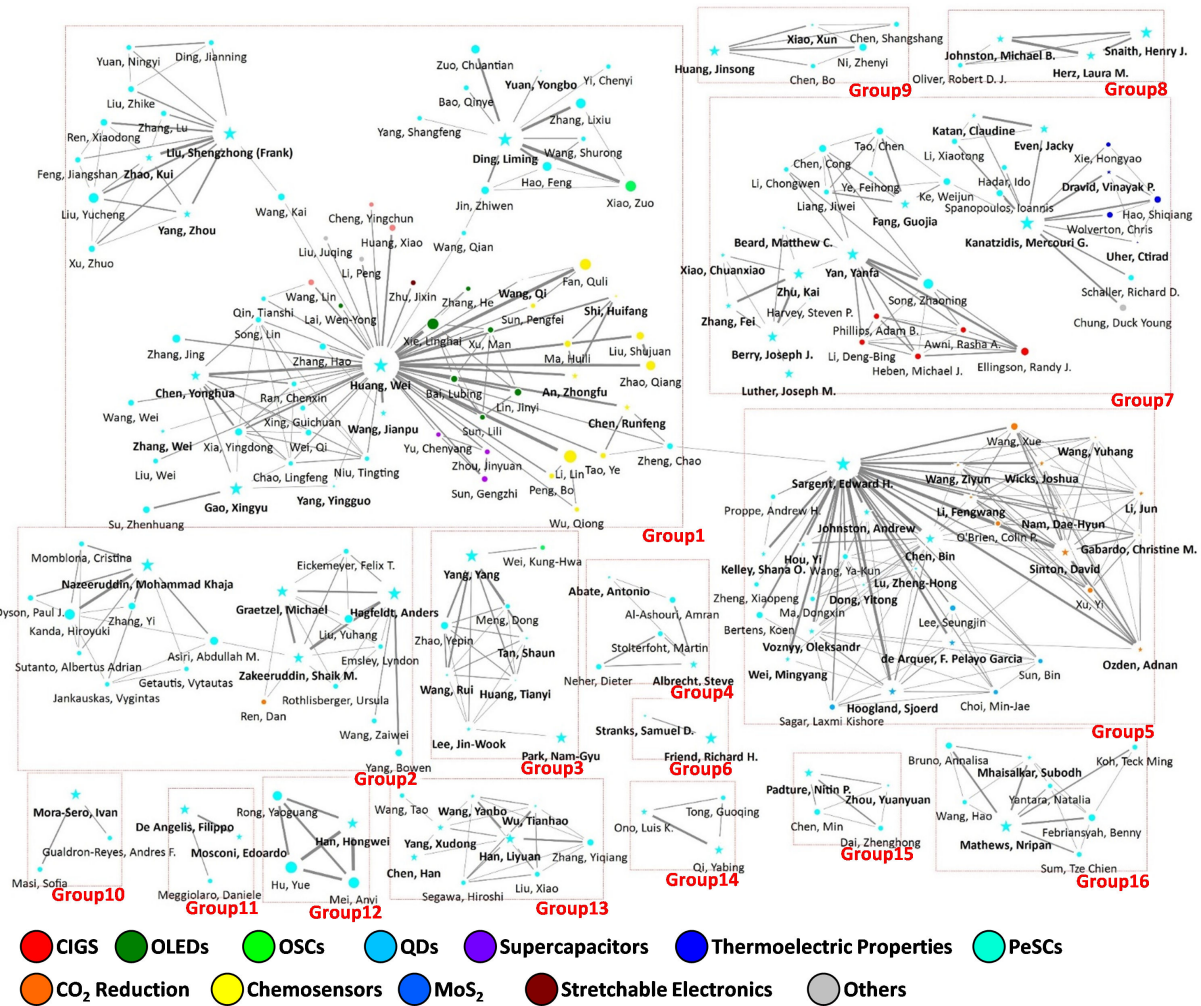
Collaboration network of researchers based on papers published during the period 2020−2022 with node colours representing respective InCites citation topics.

Figures 11 and 12 depict the resultant research collaboration networks for the 2005−2011 and 2020−2022 periods, respectively. Linkage data were obtained from paper co-authorship patterns, in which the top 3% of productive researchers in terms of publication volume are depicted. The thickness of the lines between nodes represents the intensity of the collaboration, whereas the size of each node denotes the betweenness centrality. Additionally, the colour of each node expresses the InCites citation topics of the respective researchers, providing insights into their specific areas of focus. Key metrics, such as group size, density and E–I index, were used to quantify collaboration patterns and the extent of interdisciplinary interactions within each group, as listed in tables 2 and 3. Furthermore, a representative PeSC-HCR was identified in each group, highlighting notable contributors within the collaboration networks.

**Table 2.** Group characteristics in collaboration network based on papers published during 2005−2011.

group	main research fields	group size	group density	group E–I index	representative PeSC-HCR
1	DSSCs	57	0.083	−0.985	Graetzel, Michael
2	OSCs	18	0.222	−1.000	Yang, Yang
3	DSSCs	3	1.000	−1.000	Park, Nam-Gyu
4	thermoelectric properties	20	0.211	−1.000	Kanatzidis, Mercouri G.
5	QDs	4	1.000	−1.000	Luther, Joseph M.
6	QDs	15	0.400	−1.000	Sargent, Edward H.
7	DSSCs	8	0.536	−1.000	Bisquert, Juan
8	OSCs, hyperpolarizability	18	0.379	−1.000	Jen, Alex K.-Y.
9	OSCs	24	0.149	−0.952	Friend, Richard H.
10	self-assembled monolayers	7	0.429	−1.000	Cahen, David
11	OSCs, ZnO	22	0.385	−1.000	Fang, Guojia
12	OSCs	3	1.000	−1.000	Berry, Joseph J.
13	supercapacitor	8	0.321	−1.000	Yang, Shihe

**Table 3.** Group characteristics in collaboration network based on papers published during 2020−2022.

group	main research fields	group size	group density	group E–I index	representative PeSC-HCR
**1**	PeSCs, chemosensor, OLEDs	73	0.053	−0.986	Huang, Wei
**2**	PeSCs	19	0.228	−1.000	Graetzel, Michael
**3**	PeSCs	9	0.583	−1.000	Yang, Yang
**4**	PeSCs	5	0.600	−1.000	Albrecht, Steve
**5**	PeSCs, CO_2_ reduction, QDs	32	0.276	−0.986	Sargent, Edward H.
**6**	PeSCs	2	1.000	−1.000	Friend, Richard H.
**7**	PeSCs, CIGS, thermoelectric properties	34	0.125	−1.000	Kanatzidis, Mercouri G.
**8**	PeSCs	4	1.000	−1.000	Snaith, Henry J.
**9**	PeSCs	5	0.700	−1.000	Huang, Jinsong
**10**	PeSCs	3	0.667	−1.000	Mora-Sero, Ivan
**11**	PeSCs	3	0.667	−1.000	De Angelis, Filippo
**12**	PeSCs	4	1.000	−1.000	Han, Hongwei
**13**	PeSCs	9	0.583	−1.000	Han, Liyuan
**14**	PeSCs	3	1.000	−1.000	Qi, Yabing
**15**	PeSCs	4	1.000	−1.000	Padture, Nitin P.
**16**	PeSCs	8	0.571	−1.000	Mathews, Nripan

The network nodes were clustered into 13 groups using a betweenness community analysis (figure 11); most nodes were grouped by their respective topics. The majority of groups within the research collaboration network were not interconnected, which was also confirmed by the negative group E–I index, as summarized in table 2. This segmentation probably stems from a wide array of research fields, such as DSSCs, OSCs, QDs and OLEDs. Notably, the nodes and groups related to DSSCs and OSCs constituted the largest portion of the network, underscoring their prominence during this period. Among these groups, group 1, which focused on DSSC-related nodes and was represented by Michael Graetzel, was the largest in terms of size, followed by the OSC-related groups, group 9 and group 11. In addition to the PV-oriented groups, thermoelectric and QD-related groups also had significant sizes: group 4 with a centre node of Mercouri G. Kanatzidis, and group 6 with a centre node of Edward H. Sargent. Interestingly, the large groups exhibited lower density, with central nodes having numerous direct connections to other nodes. For example, group 1 had a group size of 57 and a significantly low group density of 0.083. Michael Graetzel, the main node, was directly connected to 43 other nodes, including Sang-Il Seok, Henry J. Snaith and Michael D. McGehee. This suggests that a few highly influential nodes within these groups serve as key hubs of collaboration and knowledge transfer. In addition, geographical distance seems to play an important role in shaping collaboration patterns within the PeSC-HCR community. When visualizing the network with node colours representing researchers’ countries (electronic supplementary material, figure S2), it becomes clear that various clusters are predominantly composed of researchers from the same country or region: group 1 → EU, group 2 → USA, group 3 → South Korea, group 4 → USA, group 6 → Canada, group 9 → England and group 11 → China. This highlights the importance of geographical proximity between institutions in fostering research collaboration [19,74].

During the 2020−2022 period (figure 12), 16 distinct groups emerged. Compared with previous periods, these groups became more segmented, particularly around the highly influential PeSC-HCRs. As expected, most groups and nodes were associated with PeSCs. Notably, all PeSC-HCRs who previously focused on DSSCs or OSCs (e.g. Michael Graetzel, Henry J. Snaith, Yang and Jinsong Huang) shifted their main research area to PeSCs. This migration from established PV technologies to the emerging field of PeSCs reflects the recognition of the inherent potential of perovskite materials by the scientific community. Despite the broader diffusion of PeSC research focus within the PeSC-HCR community, the collaborative relationships influenced by geographical distance remained consistent with those observed during the 2005−2011 period, as shown in electronic supplementary material, figure S3.

In large groups, the nodes represented a combination of other research domains. Group 1, led by Wei Huang with a group size of 73, covers a range of areas, such as OLEDs, chemosensors and supercapacitors. Similarly, group 5, led by Edward H. Sargent with a group size of 32, consisted of nodes related to CO_2_ reduction and QDs. In addition, group 7, led by Mercouri G. Kanatzidis and Yanfa Yan with a group size of 34, was connected to the nodes related to thermoelectrics and CIGS. These phenomena appear to stem from the conventional research focusing on PeSC-HCRs within these groups. For instance, in previous periods, Wei Huang was involved in OLEDs and chemosensors, and Edward H. Sargent was involved in QDs. Interdisciplinary collaborations within these groups should not necessarily be interpreted as researchers transitioning to entirely different domains. Rather, it is more reasonable to view this as an extension of their ongoing research, reflecting both the continuity of their previous work and the integration of their expertise into the broader field of PeSC research.

Similar to the 2005−2011 period, there was a lack of extensive collaboration between different groups, and network density was relatively low. Although research on PeSCs has been intensively activated and most of the nodes have become related to PeSCs, collaborations among PeSC-HCRs have been primarily confined to the initial clusters established before the advent of PeSCs rather than expanding to the entire research network. Primarily, the influence of research areas and collaborative relationships before the advent of the PeSC field can significantly affect current research collaboration patterns. Moreover, the PeSC-HCRs, who were influential before the emergence of PeSCs, continued to hold significant positions within the collaborative network during the 2020−2022 period. This suggests that the research directions of these influential researchers have a substantial impact on the overall collaboration network and that the evolution of research themes within the PeSC-related community is heavily shaped by these key individuals.

## 4. Conclusion

Using bibliometric approaches, we explored the historical evolution of PeSC research based on the footprints of PeSC-HCRs. The key findings from our comprehensive analysis are as follows: (i) the citation relationship analysis revealed that early PeSC publications (2009−2014) predominantly cited papers on next-generation PVs. However, in the mature phase of PeSC research (2020−2022), PeSC publications were cited by papers from a wide range of scientific domains. (ii) Examination of publication volume according to research fields related to PeSCs showed a significant shift in focus around 2014. Prior to 2014, the majority of publications were on OSCs and DSSCs. After 2014, there was a sharp increase in the number of publications on PeSCs, while publications on CIGS, OSCs and DSSCs declined. (iii) PeSC-HCP analysis indicated that while the proportion of efficiency-focused HCPs has recently decreased, the proportions related to stability and public acceptability have increased significantly. (iv) Keyword network analysis demonstrated that prior to the widespread focus on PeSCs, PeSC-HCRs explored diverse areas. From 2015 to 2017, research efforts focused on PeSC stability, which broadened in 2020−2022 to encompass Pb-free materials, scalability and other perovskite applications. (v) Collaboration network analysis revealed that collaborations among PeSC-HCRs were largely confined to initial clusters established before the advent of PeSCs. PeSC-HCRs, who were influential prior to the rise of PeSCs, continued to hold significant positions within the collaboration network during the 2020−2022 period.

These comprehensive findings suggest that with the advent of PeSCs, researchers from various disciplines, previously focused on novel PV materials, have increasingly shifted their efforts towards PeSC research. While concentrating on PeSC studies, many of these researchers are also expanding their work into other perovskite-based applications. As device efficiency has improved and the technology has matured, research areas, such as stability, Pb-free materials and scaling-up, have become key to successful commercialization and market deployment of PeSCs. Moreover, emerging applications in memristors, piezoelectric devices and X-ray imaging underscore the broad potential of perovskite materials. Simultaneously, existing collaborative networks and influential researchers continue to guide the research direction throughout this knowledge transfer process.

To the best of our knowledge, this is the first study to provide a holistic view of PeSC research trends using bibliometric analysis. Our systematic approach sheds light on the interdisciplinary nature and intellectual framework of PeSC studies and forecasts potential future technological directions, marking a significant contribution to both the quantitative and qualitative understanding of the field.

One limitation of this study is that the data extend to 2022. As PeSC-HCR research continues to evolve, incorporating data from 2023 to 2025 would provide additional insights and help track emerging trends and shifts in focus. Furthermore, this study is limited by its exclusive focus on PeSC-HCRs, which may overlook the contributions of other prominent researchers who have not yet reached high citation thresholds. Expanding the scope to include a broader range of researchers in future analyses would provide a more comprehensive understanding of the PeSC research landscape.

## Data Availability

In line with open science principles, we have uploaded the dataset to Dryad [75]. Supplementary material is available online [76].
